# Inorganic Nanozymes: Prospects for Disease Treatments and Detection Applications

**DOI:** 10.3389/fchem.2021.773285

**Published:** 2021-11-25

**Authors:** Peng Wang, Dongyu Min, Guoyou Chen, Minghui Li, Liquan Tong, Yonggang Cao

**Affiliations:** ^1^ Harbin Medical University-Daqing, Daqing, China; ^2^ The Affiliated Hospital of Liaoning University of Traditional Chinese Medicine, Shenyang, China; ^3^ Department of General Surgery, The Fifth Affiliated Hospital of Harbin Medical University, Daqing, China

**Keywords:** nanozyme, catalytic mechanisms, tumor therapy, anti-inflammatory, biosensing

## Abstract

In recent years, with the development of nanomaterials, a slice of nanomaterials has been demonstrated to possess high catalytic activity similar to natural enzymes and counter the dilemmas including easy inactivation and low yield natural of enzymes, which are labeled as nanozymes. The catalytic activity of nanozymes could be easily regulated by size, structure, surface modification and other factors. In comparison with natural enzymes, nanozymes featured with a more stable structure, economical preparation and preservation, diversity of functions and adjustable catalytic activity, thus becoming the potentially ideal substitute for natural enzymes. Generally, the are mainly three types containing metal oxide nanozymes, noble metal nanozymes and carbon-based nanozymes, owing various applications in biomedical, energy and environmental fields. In this review, to summarize the recent representative applications of nanozymes, and potentially explore the scientific problems in this field at the same time, we are going to discuss the catalytic mechanisms of diverse nanozymes, with the emphasis on their applications in the fields of tumor therapy, anti-inflammatory and biosensing, hoping to help and guide the future development of nanozymes.

## Introduction

Enzymes are proteins or RNAs generated by living cells that are specific to their substrates with highly catalytic, who plays a key role in significant class of biocatalysts. With high catalytic efficiency and strong selectivity, enzymes have been widely utilized in disease diagnosis, biosensing and environmental protection (Chen and Arnold, 2020). However, natural enzymes are trapped of easing to denaturation under extreme conditions, high extraction cost and relative low utilization, which make it formidable to be applied in large-scale industrial production. Therefore, it is highly desirable and essential to find a natural enzyme substitute with strong and stable catalytic activity. In this case, inorganic nanozymes act as an emerging star has captured people’s attention in recent decades (Wei and Wang, 2013). As early as 2007, Chinese scientists first discovered that Fe_3_O_4_ nanoparticles possess horseradish like properties, whose catalytic mechanism and efficiency are similar to horseradish peroxidase peroxidase (HRP) (Gao et al., 2007). This research breaks the long-held belief that inorganic materials are bioinert materials and opens the field of nanozyme research. Up to now, 540 kinds of nanomaterials have been reported to own enzyme-like activities (Wu et al., 2019; Wang L et al.,2019), including a total of 50 elements. Among them, 117 kinds of nanozymes have been utilized for disease treatments. So far, nanomaterials have been found to possess a variety of oxidoreductase activities, containing peroxidase (POD) (Li L et al., 2021; Li X et al., 2021; Liu C et al., 2021; Su et al., 2021; Zhang J et al., 2021), catalase (CAT) (Jia et al., 2019; Liu et al., 2021a; Wu et al., 2021; Xu et al., 2021; Zhang Y et al., 2021), superoxide dismutase (SOD) (Liu et al., 2021b; Liang and Yan, 2019; Lv et al., 2021; Sang et al., 2020; Yuan et al., 2021), oxidase (OXD) (Hu et al., 2021; Lin et al., 2021; Liu Y et al., 2021; Zhang J et al., 2021; Zhu et al., 2021) and glutathione peroxidase (GPx) etc. (Gao and Yan, 2013). The catalytic activity of nanozymes can be adjusted by changing size, structure, composition and surface modification of their nanomaterials (Meng et al., 2019). According to their components, nanozyme could be divided into metal oxide nanozyme, noble metal nanozyme and carbon-based nanozyme. This article will introduce the latest research progress of nanozyme the treatment of diseases from three therapeutic strategies. We hope to summarize the research situation of nanozyme in biomedical application (Table 1), so as to clarify the bottleneck problems in laboratory research and clinical transformation of nanozyme, making prospects for the future development of nanozyme.

**TABLE 1:** Types and applications of nanozymes.

Nanomaterials	Enzyme activity	Applications	Ref.	*K*_M_	*V*_max_
PEG/Ce-Bi@DMSN	POD/CAT	Catalytic oxidation for cancer therapy	21	27.54 mM	3.85 × 10^-8^ M/s
Fe_3_O_4_@DMSN	POD	Hydroxyl radical generation for cancer therapy	27	10.93 mM	4.22 × 10^-8^ M/s
V_2_O_5_	GPx/GR	Biological special applications	31	V_Nw_, (44.4 ± 1.7), V_Sh_, (57.3 ± 3.8), V_Nf_ (92.5 ± 3.4), and V_Sp_ (143.7 ± 2.3),	V_Nw_,(192.3 ± 6.6), V_Sh_, (233.1 ± 16.3), V_Nf_ (340.1 ± 21.3), and V_Sp_ (458.7 ± 19.6),
Pd-ZIF	POD/GPx	Ferroptosis for cancer therapy	39	0.24 mM	1.97 × 10^-5^ M/s
Co_3_O_4_@Co-Fe	POD	Biosensing and dye-degradation	44	0.176 (k/min)	3.93 (t_1/2_)
Fe_3_O_4_-Graphene	SOD	Glucose detection	45	1.39 mM	10.1 × 10^-8^ M/s
N-PCNSs	POD/OXD/CAT/SOD	Catalytic oxidation for cancer therapy	54	0.1540 M	0.4720 mg/L/min
MSN-AuPs	POD/OXD	Anti-bacterials	56	15.81 mM	17.3 × 10^-8^ M/s
Cu-TCPP	POD/GOX	Anti-bacterials	57	88–90% Inactivation of •OH	80–100% Catalytic activity
IONzymes	SOD	Anti-bacterials	58	259.7 mM	96.4 nM/s
I-CDs	POD	Antifungal activity	59	0.097 mM	27.86 × 10^-8^ M/s
Mn_3_O_4_	SOD	Anti-inflammatory	62	75% scavenging rate	/
Ce_0.7_Zr_0.3_O_2_	SOD	Anti-inflammatory	63	/	/
MPBZs	SOD	Anti-inflammatory for colitis	64	/	/
PB	SOD/CAT	Anti-inflammatory	66	14.7 mM	1.15 × 10^-7^ M/s

## The Construction of Nanozymes

### Metal Oxide Enzymes

**FIGURE 1:**
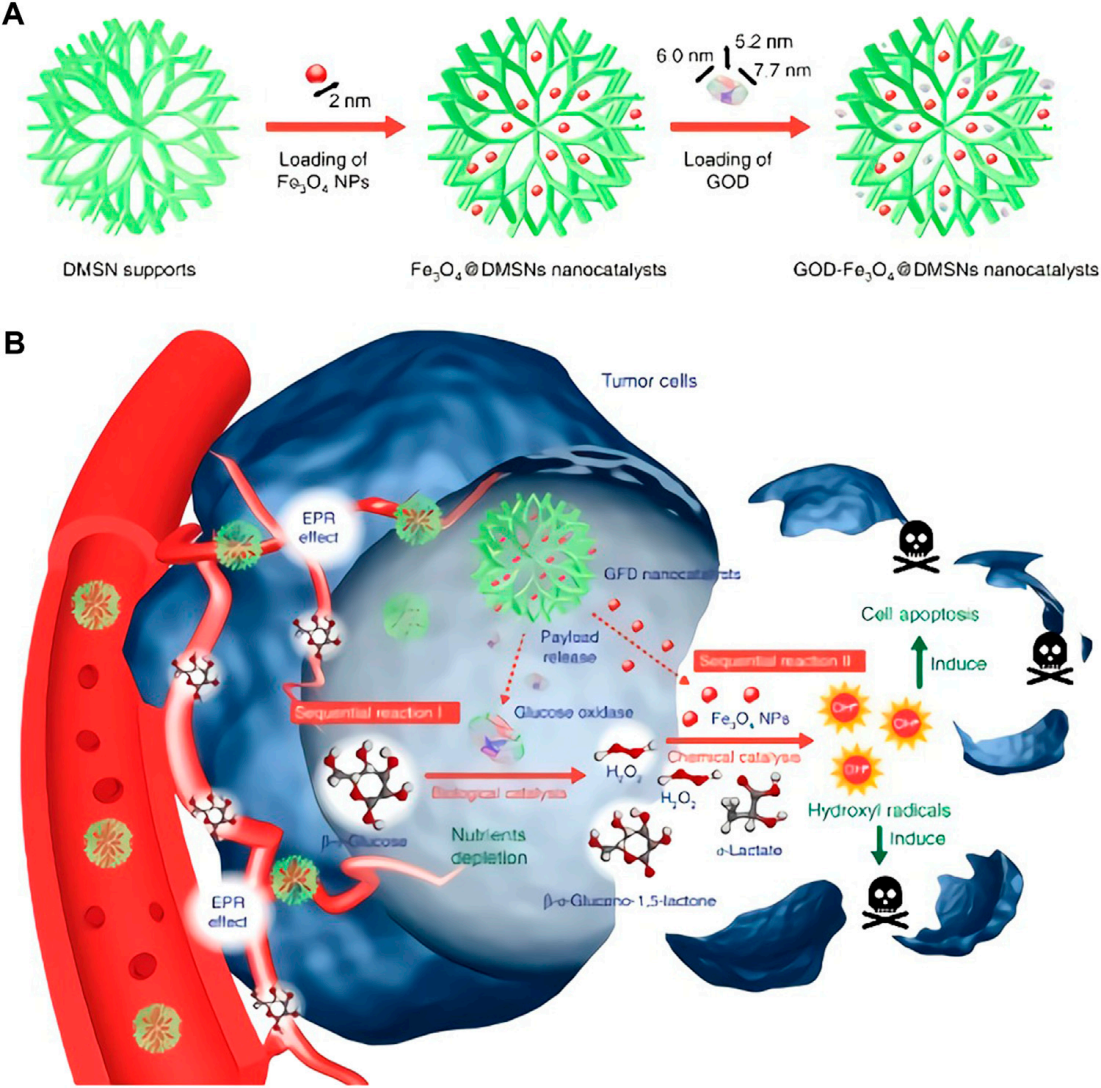
Fabrication and catalytic-therapeutic schematics of sequential GFD NCs. **(A)** Synthetic procedure for Fe_3_O_4_@DMSNs nanocatalysts and GOD-Fe_3_O_4_@DMSNs nanocatalysts. The sizes of the prepared Fe_3_O_4_ nanoparticles and adopted GOD are indicated in the scheme. **(B)** The scheme of sequential catalytic-therapeutic mechanism of GFD NCs on the generation of hydroxyl radicals for cancer therapy. (a,b was reproduced with permission from Ref. (Huo et al., 2017). Copyright 2017 Nature Publishing Group.)

Transition metals featured with variable valence and excellent stability, making them superior candidates for inorganic enzymes. The catalytic process of nanozymes involves electron transfer and valence changes, indicating oxidoreductase activity. Fe_3_O_4_ nanoparticles was first found to possess POD activity. The co-existence of ferric and ferrous ions endows it with superior POD and CAT properties (Figure 1) (Huo et al., 2017). It has been exploited that under acidic conditions, through the typical Fenton reaction, Fe_3_O_4_ could react with hydrogen peroxide to produce a large number of highly ·OH. Upon neutral or alkaline conditions, it will promote the decomposition of H_2_O_2_ to produce O_2_. The reason is that H_2_O_2_ is more inclined to decompose into H_2_O and O_2_ at higher pH, while the Fe^3+^ in Fe_3_O_4_ just possess excellent catalytic ability, thus accelerating the reaction (Chen et al., 2012). What is similar to Fe_3_O_4_ are cerium oxide nanoparticles, at the neutral pH value, the content of cerium in the oxidation state +4 was higher, and the cerium oxide presented more significant CAT activity. However, at acidic pH and higher level of cerium atom on the surface of oxidation state +3, cerium oxide presented reduced CAT activity, while its POD activity was more active, which can catalyze the reaction of TMB in the absence of H_2_O_2_ (Asati et al., 2009; Jiao et al., 2012). In addition, nanozyme with GPx properties have also aroused great attention. Ghosh et al. designed an active V_2_O_5_ nanozyme with redox properties and similar to GPx in four different forms. They demonstrated that the POD simulated activity of four orthogonal V_2_O_5_ nanozymes with different forms. Nanowire (VN_w_), nanosheets (VS_h_), nanoflowers (VN_f_), and nanospheres (VS_p_) can adjust the redox reaction by adjusting the size, shape and crystal plane, indicating that the activity is independent of their surface area, which provides an opportunity for the design and synthesis of nanomaterials with enzyme-like activity (Ghosh et al., 2018).

### Noble Metal Nanozyme

**FIGURE 2:**
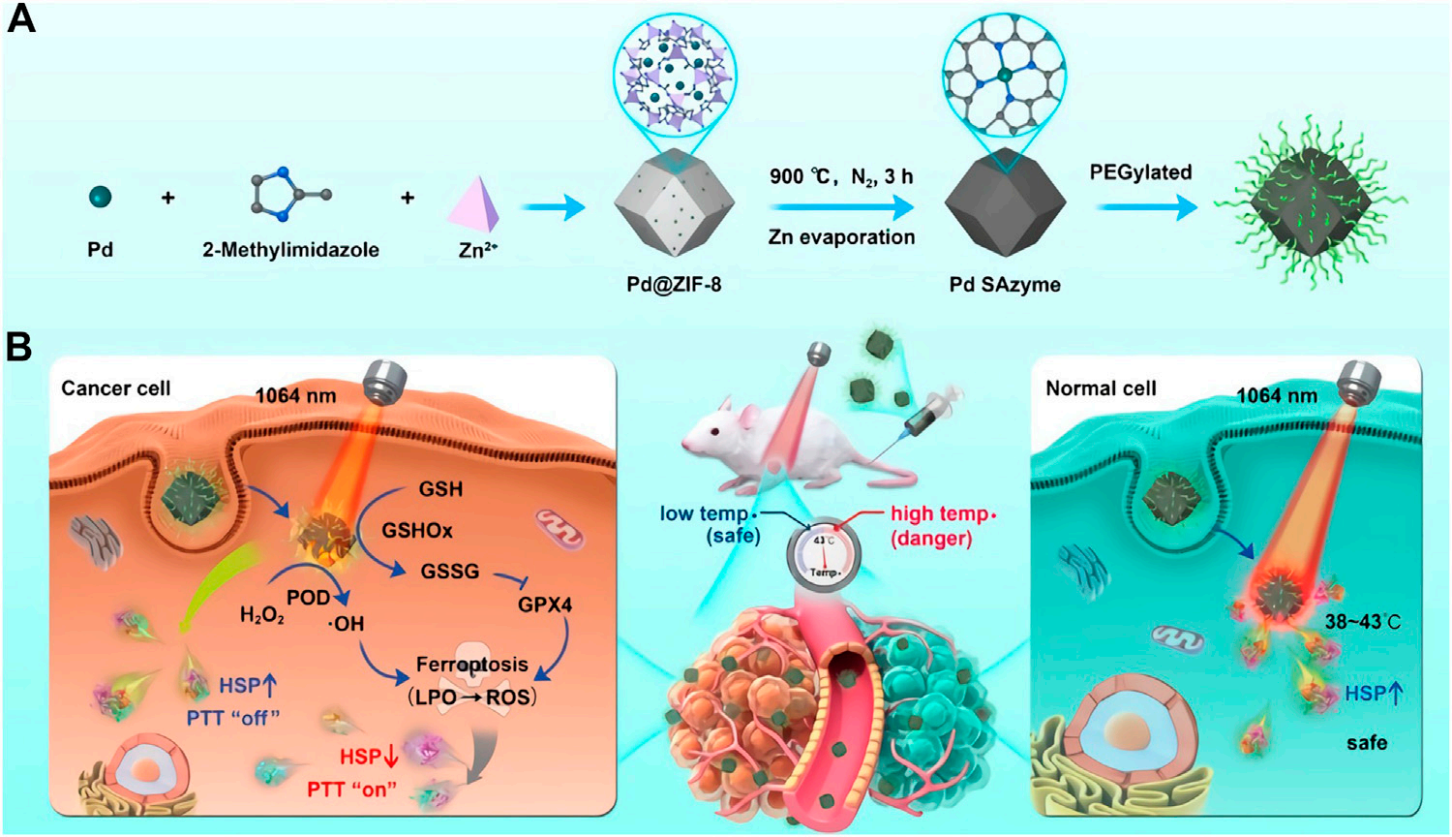
Schematic illustration of the formation of PEGylated Pd SAzyme **(A)** and the mechanism of ferroptosis promoted by mild PTT **(B)**. (**A,B** was reproduced with permission from Ref. (Chang et al., 2021). Copyright 2021 John Wiley and Sons.).

Noble metal based nanozymes possess special optical properties, excellent chemical stability, adjustable enzyme-like activity and superior biocompatibility, which are the hot research materials in the field of nanobiology and medicine. Among them, Au and Pt nanozymes possess POD (Jv et al., 2010), SOD and CAT activities (He et al., 2013). Besides, Au quantum dots also possess glucose oxidase (GOD) activity, and experiments results demonstrated that the activity of GOD was negatively correlated with particle size, for the smaller the particle size presents the higher the GOD activity, which due to the fact that small particle sizes own larger surface area (Comotti et al., 2004). Different from the catalytic mechanism of electron transfer of transition metal ions, noble metal nanozymes are generally stable and no valence changes occur in the catalytic reaction, and SOD activity is due to thermodynamics and kinetics. Taking Au (111) surface as an example, under acidic or neutral conditions, H_2_O_2_ is adsorbed on Au (111) surface and a basic decomposition reaction occurs, resulting in the adsorption of oxygen species O* (*means adsorption on the surface of the metal material). O* can take hydrogen atoms make its oxidation reaction of organic substrates, presenting the POD activity. Under alkaline conditions, hydroxyl groups are pre-adsorbed on Au (111) surfaces. Under the action of OH*, H_2_O_2_* occurs acid decomposition reaction, generating oxygen adsorption species O_2_*. O_2_* is released as O_2_ after separating from the metal surface, showing CAT-like activity. Among them, OH* is both an active site like CAT and an inhibitory site of POD activity (Sun et al., 2015). When pH increases, H_2_O_2_* is more prone to occurs acid decomposition, leading to the decrease of POD-like activity and the promotion of CAT activity (Li J et al., 2015). Theoretical calculation also revealed that the greater the adsorption energy of hydrogen peroxide on the metal surface, the higher the catalytic activity of the metal, which provides a basis for rational design of nanozymes. Common noble metal nanozymes also contain Pd and Ag nanozymes, which own POD activity (Fu et al., 2015; Wen at al., 2015). Under neutral and alkaline conditions, Pd nanozymes exhibit CAT and SOD activities. Less reported, the single atomic nanozyme formed by Pd attached to ZIF-8 has the dual functions of GPx and POD, which can convert GSH to GSSG when it reacts with H_2_O_2_ to generate ·OH, thus achieving a variety of functions (Figure 2) (Chang et al., 2021).

### Carbon-Based Nanozymes

Carbon-based nanomaterials, mainly include carbon nanotubes, graphene oxide, carbon nitrous oxide, carbon dots, fullerenes and their derivatives, have been broadly explored to mimic enzyme-like activities. The categorical electronic and geometric structures endure carbon-based nanozymes with various enzyme activities. In fact, carbon-based nanozymes hold catalytic properties similar to those of natural enzymes and have been mentioned in early studies. As early as 1991, Fulleren for the first time was found to own the ability to scavenge free radicals (Krusic et al., 1991), but at that time, natural fullerenes were insoluble in water and were not widely concerned. Subsequently, graphene as a two-dimensional carbon-based nanomaterial has aroused a lot of attention. Ren et al. designed a graphene oxide quantum dot with excellent CAT activity that effectively reduced Reactive oxygen species (ROS) content in PC12 cells (Ren et al., 2018). Even at low concentrations, its catalytic capacity is comparable to CAT (4U/mL), making it an excellent enzyme substitute. In 2018, Li et al. reported of Co and N doped into porous car nanozyme (Co, N-HPC), which presenting excellent OXD activity (Li et al., 2018). They found that the pyridine N and pyrrole N atoms increased the electron state density and opened the band gap of graphene, which effectively promoted the electron transfer of nanozyme from catalyst to O_2_, thus enhancing the catalytic performance of graphene. Based on carbon-based nanozymes generally possessing POD activity, (Sun et al., 2018) reported the latest progress of this research group on the catalytic mechanism of carbon-based nanomaterials in 2010, and proposed that the POD activity of carbon-based nanomaterials lies in the carbonyl group (C=O) on the surface as the active site, and the carboxyl group (--COO−) as the binding site, which binds to H_2_O_2_ molecule and converts it into ∙OH. With the further exploration, carbon-based nanozymes will be applied in biological diagnostics and biosensing.

## Applications

### Nanozyme for Biosensing

**FIGURE 3:**
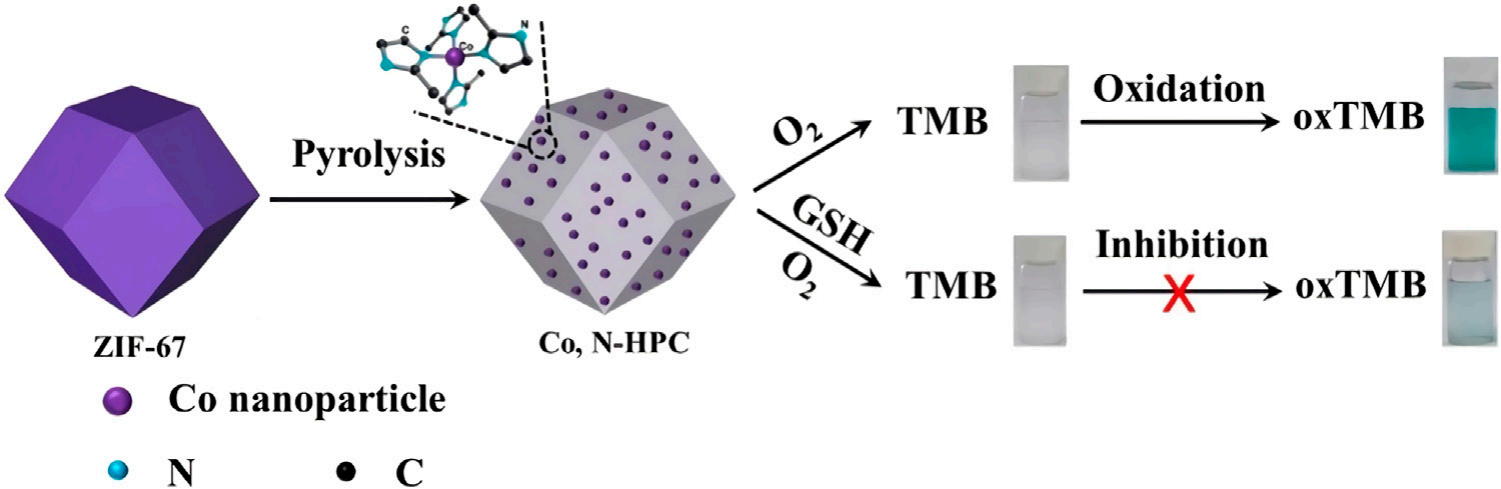
The schematic diagram of Co_3_O_4_@Co-Fe hybrids was reproduced with permission from Ref. (Chen H et al., 2020). Copyright 2020 Elsevier.

**FIGURE 4:**
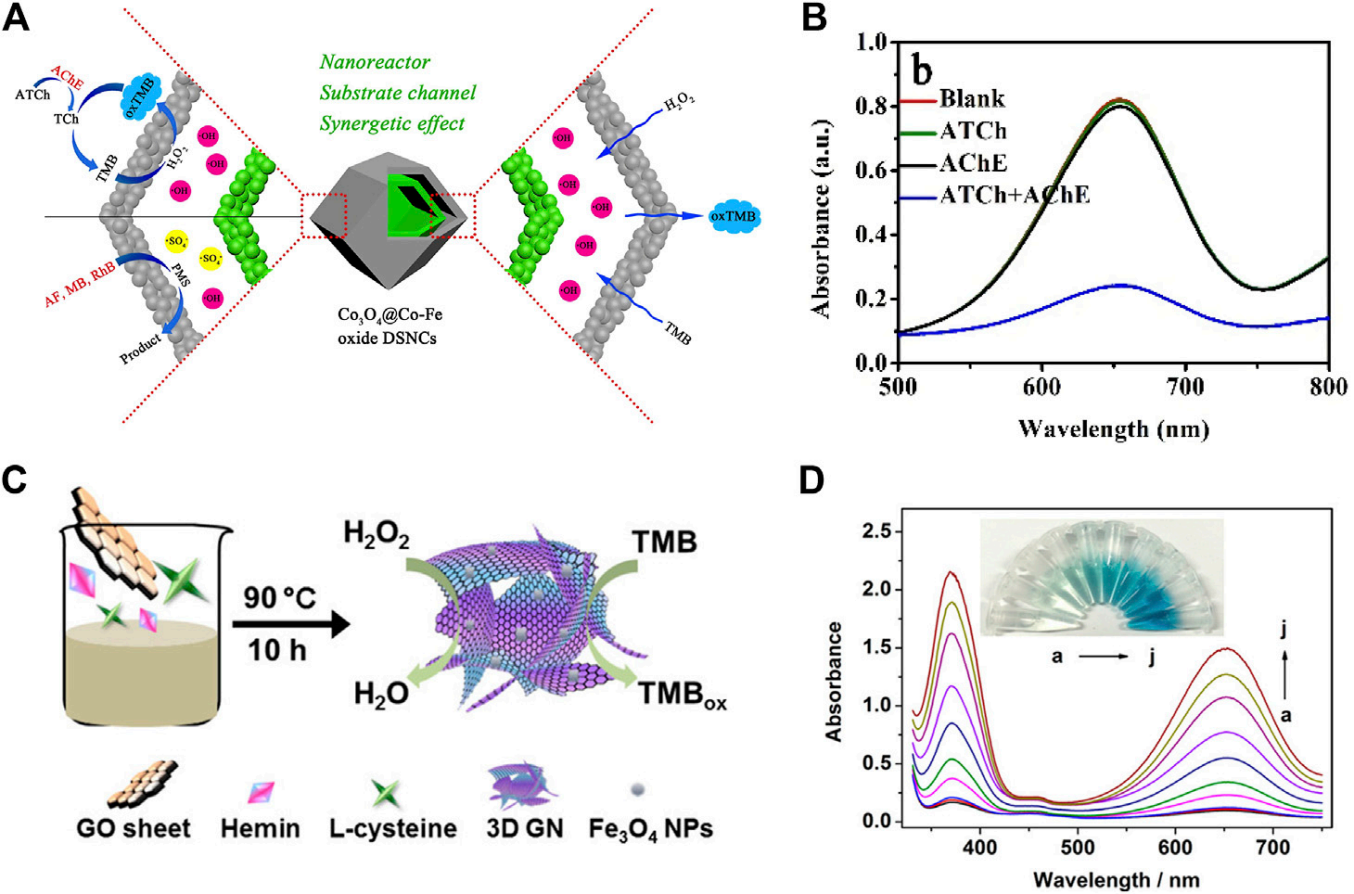
The schematic diagram of Co_3_O_4_@Co-Fe hybrids **(A)**. UV–vis spectra of Co_3_O_4_@Co-Fe oxide DSNCs/H_2_O_2_/TMB system with 0.25 mM ATCh, 1 mU mL^−1^ AChE, or 0.25 mM ATCh + 1 mU mL^−1^ AChE **(B)**. Schematic Presentation for Fe_3_O_4_ NPs Loaded 3D Graphene Nanocomposites with POD-Like Activity **(C)**; Absorption spectra of TMB with different glucose concentrations: 0, 0.005, 0.01, 0.05, 0.1, 0.2, 0.3, 0.5, 0.7, and 1.0 mM **(D)**. (**A,B** was reproduced with permission from Ref. (Chen Q et al., 2020). Copyright 2020 Elsevier. **(C–D)** was reproduced with permission from Ref. (Wang et al., 2017). Copyright 2017 American Chemistry Society).

The detection of glucose in plasma is always of great significance for biosensing. On basis of the catalytic activity of nanozyme and the principle of glucose oxidation producing H_2_O_2_, the colorimetric detection method of H_2_O_2_ and glucose is expected to be utilized in practical application. At present, increasing studies have been reported on the application of nanozymes in biosensors, among which the most popular is the detection of H_2_O_2_ and glucose. Huang et al. devised Co_3_O_4_@Co-Fe oxide double-shell nanocages (DSNCS) by anion exchange combined with low temperature pyrolysis utilizing ZIF-67 as the starting template (Figure 3, Figures 4A,B) (Chen H et al., 2020). The nanocages can maximize the advantage of hollow nanostructures that act as both nanoreactors and substrate channels to mimic enzymes. The excellent POD-like activity of Co_3_O_4_@Co-Fe oxide DSNC endures the linear detection with range of H_2_O_2_ from 0.02 to 600 μM, with the detection limit was 20 nM. Furthermore, Co_3_O_4_@Co-Fe oxide DSNC can activate PMS to degrade 99.1% of acidic fuchsin in 20 min, and possess superior reusability in ten cycles of operation. Taking advantages of high POD activity of Graphene quantum dots (GQD), (Figures 4C,D) (Wang et al., 2017) designed a novel one-pan strategy to prepare 3D porous graphene (3D GN) loaded with Fe_3_O_4_ nanoparticles employing heme as the iron source. The obtained product (3D GH-5) presented superior POD activity. In comparison with the traditional 2D graphene-based nanocomposites, the introduced 3D porous structure significantly improved the catalytic activity, catalytic rate and affinity for the substrate. The high catalytic activity can be attributed to the formation of Fe_3_O_4_ NPs and the 3D porous graphene structure.

Nanozymes were taken advantages of glucose detection when combined with GOD. GOD is able to catalyze the oxidation of glucose into gluconic acid and H_2_O_2_, and then nanozymes are utilized to detect H_2_O_2_ to obtain the glucose concentration indirectly (Wei and Wang, 2008). Zhang et al. reported a 3D Fe- and N-doped carbon structure (Fe-Phen-CFS) (Zhang et al., 2015), which acts as a POD-mimic nanozyme for the detection of H_2_O_2_ and glucose. In the existence of H_2_O_2_, Fe-Phen-CFS is able to catalyze the conversion of the substrate to the fluorescence product of hydroxyl terzoic acid, realizing the quantitative detection of H_2_O_2_. The detection limitation is approximately 68 nM, and the linear range is 0.1–100 mM.

The application of nanozyme in biosensing also includes nucleic acid detection. Park et al. (2011) performed polymerase chain reaction (PCR) on the samples. If target DNA is present in the sample, a large amount of nucleic acid is produced and mixed with iron oxide nanoparticles to develop color. Through electrostatic interaction, the phosphoric acid skeleton will be adsorbed on the surface of the nanoparticles, resulting in the binding of the chromogenic substrate to the nano-enzyme for the detection of the target DNA. Similarly, in human urine Papillomavirus DNA (Brinkman et al., 2004) and *Chlamydia trachomatis* (Hafner et al., 2008) can also be detected by nanozyme. Wang et al. (2013) loaded ferroporphyrin and *Streptomyces* anti-biotin proteins onto nanozymes for electrochemical detection of target DNA. The hairpin DNA was fixed on the electrode modified by Au NPs nanozyme. When the target DNA was present in the sample, the hairpin DNA was opened and *Streptomyces* anti-biotin protein loaded on the nanozyme was bound to the biotin. In addition, aptamer can be combined with nano-enzyme to capture target protein, which is expected to replace the traditional detection of protein by antibody Methods. From what has been discussed above, the advantages such as high sensitivity, wide detection range and broad spectrum durability of organic nanozyme make it an important detection method for biosensing, which is expected to replace traditional detection methods using natural enzymes.

### Tumor Therapy

Cancer is a global public health problem which endangers human life and health. (Erratum, 2020). There are growing methods for tumor therapies including chemotherapy, radiation therapy, photodynamic therapy, sonodynamic therapy and immunotherapy treatments. However, chemotherapy and radiotherapy always bring serious side effects, while O_2_-dependent photodynamic and sonodynamic therapy are difficult to cope with the hypoxic environment of tumors, which limit their applications in tumor suppression. Nanozymes are featured with various enzymatic activity, which not only can induce tumor cells apoptosis by catalyzing the generation of ROS storm, but also promote the generation of O_2_ to relieve the hypoxia tumor microenvironment, which making it a significant role in tumor treatment.

**FIGURE 5:**
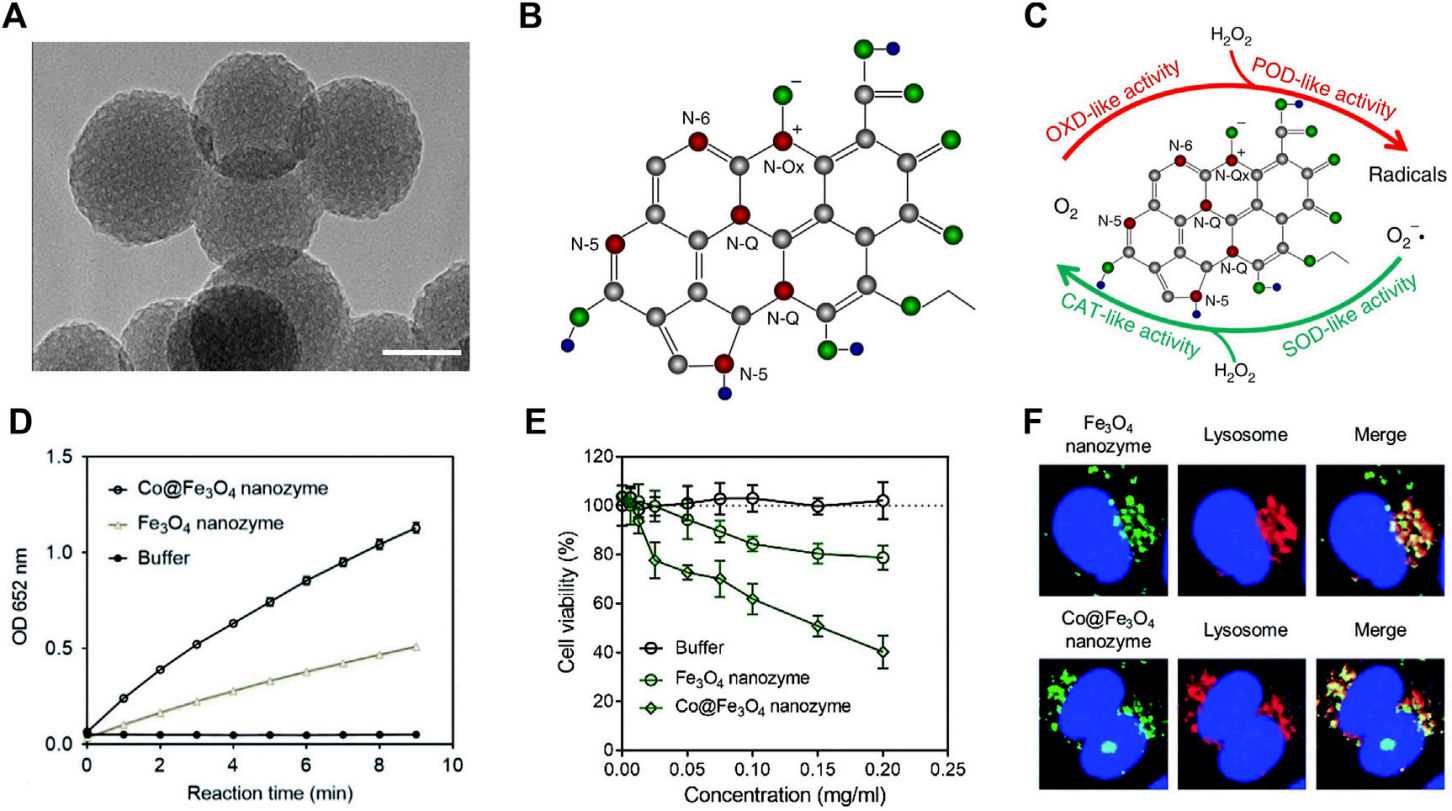
Kinetics for OXD-like activity of N-PCNSs **(A)** Kinetics for POD-like activity of N-PCNSs **(B)**. Schematic presentation of enzyme-like activities of N-PCNSs **(C)**. Comparison between the POD-like activities of the Fe_3_O_4_ and Co@Fe_3_O_4_ nanozymes **(D)**. Cell viability of the human renal cancer cells A-498 incubated with the Fe_3_O_4_ and Co@Fe_3_O_4_ nanozymes. The buffer contained 10 nM H_2_O_2_ **(E)**. Localization of the Fe_3_O_4_ and Co@Fe_3_O_4_ nanozymes in the A-498 cells **(F)**. (**A–C** was reproduced with permission from Ref. (Fan et al., 2018). Copyright 2018 Nature Publishing Group. **(D–F)** was reproduced with permission from Ref. (Wang L et al., 2019). Copyright 2019 Royal Society of Chemistry).

Quite a few nanozymes are able to exhibit POD and OXD activities in the acidic microenvironment of tumors (Figures 5D–F) (Wang L et al., 2019). They were endured with strong oxidizing ability and can catalyze endogenous H_2_O_2_ or O_2_ to produce a large number of free radicals to against tumor cells. Yan et al. prepared nitrogen-doped porous carbon nanospheres with 4 types of enzyme activities (OXD, POD, CAT and SOD) (Figures 5A–C) (Fan et al., 2018). The nanozyme possess POD and OXD activity under acid environments. While under neutral conditions, the nanozyme behave CAT and SOD activities, thus it has no obvious side effect on normal cells. In a tumor-bearing mice with diverse groups, the nanozyme treatment group significantly inhibited the growth of tumors, indicating the feasibility to employ nanozymes to suppress tumors. In another similar study, (Dong et al., 2020) designed Bi macroporous silicon loaded cerium oxide quantum dots (PEG/Ce-Bi@DMSN) to treat tumors depend on the POD and CAT and GPx activities, under neutral environment, the synthesized nanozyme can catalyze the decomposition of H_2_O_2_, while at acid environment, the POD and GPx activities endure nanozyme with OH generation and GSH depletion, triggering the cell apoptosis. The nanocatalytic enzyme platform provides a new conceptual framework for the treatment of tumors.

### Antibacterial

Bacterial infection has always been one of the major problems threatening human health. To date, a number of drugs or materials such as metal ions, quaternary ammonium ions, antibiotics and fungicides have been used to inhibit or reduce the growth of harmful bacteria. However, antibiotic resistance, complex production process and high cost hampers the application of traditional sterilizers. Similar to tumor therapy, the POD activity of nanozyme that catalyze H_2_O_2_ or O_2_ to produce a large number of ROS can achieve sterilization effect. At the same time, taking advantages of high stability, broad spectrum and low cost, nanozyme is expected to be a nova of sterilizer.

**FIGURE 6:**
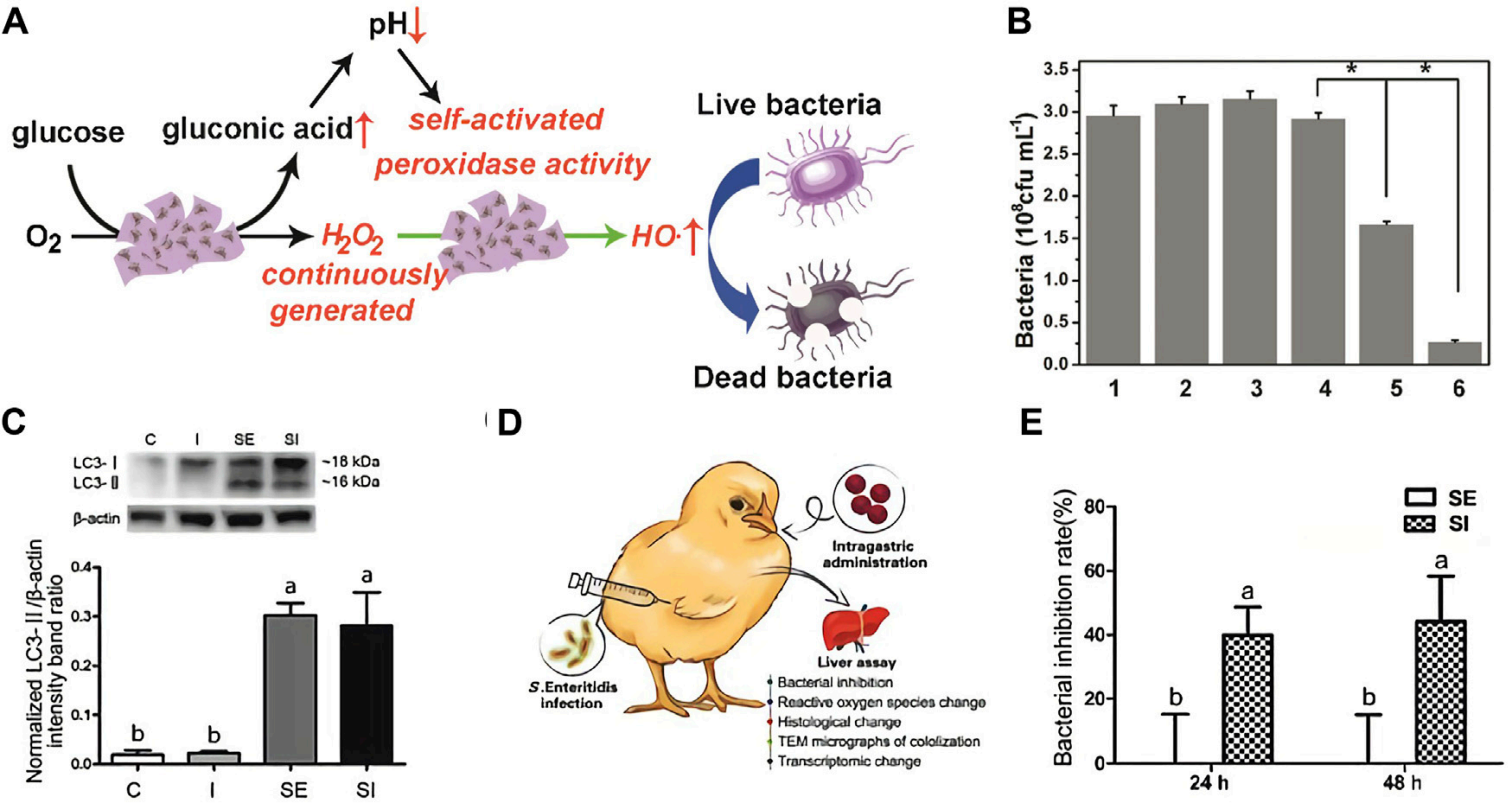
The antibacterial mechanism of the 2D MOF/GOx hybrid nanocatalyst as a benign and self-activated cascade reagent **(A)**. Viability analyses of *S. aureus* *Significantly different (*p* < 0.05) from data obtained. **(B)**. The protein expression levels of LC3, a marker protein for autophagy, in LMH cells at 12 h **(C)**. Schematic illustration of the animal experiment. Chicken were orally gavaged with ionzymes and subcutaneous injected with S. Enteritidis. Livers were collected for pathological analyses **(D)**. Bacterial inhibition rates of ionzymes at different times in livers of S. Enteritidis infected chickens. Values represent the Mean ± SEM (*n* = 9). Different letters indicate statistically significant difference (*p* < 0.05) **(E)**. (**A–C** was reported with permission from Ref. (Liu et al., 2019) Copyright 2019 American Chemistry Society. **D–E** was reported with permission from Ref. (Shi et al., 2018) Copyright 2018 Ivyspring International.)

Qu et al. (Tao et al., 2015) constructed bifunctional mesoporous silica supported gold nanoparticles (MSN-AuPs), which possess both POD and OXD enzyme activities, and can be acted as a highly efficient ROS generating agent to kill bacteria. Moreover, since the ROS produced won excellent oxidative activity to nucleic acids, proteins and polysaccharides in the biofilm matrix, the antibacterial system can effectively break the existing biofilm while sterilizing, which prevent the formation of new biofilms. Most nanozymes with POD activity operate best in an acidic environment, which severely limits their application in neutral living systems. To break the limitation, Qu (Figure 6A–C) (Liu et al., 2019) synthesis and characterization of 2D Cu-TCPP(Fe) nanocrystal based on MOF and supported by GOD. Glucose can be converted to gluconic acid and H_2_O_2_ by GOD activity *in vivo*, and the resulting gluconic acid can reduce the microenvironment pH from 7 to 3–4, which significantly activate the POD activity of 2D Cu-TCPP(Fe) nanochips, leading to the generation of boosting •OH. Moreover, Gao group (Figures 6D,E) (Shi et al., 2018) employed ferric oxide nanozymes against intracellular *Salmonella* enteritidis. The peroxide-like activity of ferric oxide nanozymes significantly suppressed the activity of *Salmonella* enteritidis in cells, and then joint with the autophagy function of cells to kill bacteria.

Furthermore, Gao’s team devised an antiviral strategy that utilized iron oxide nanozyme (ionzymes) to target the lipid envelope of influenza virus (Qin et al., 2019). They evaluated the antiviral activity of ionzymes by applying hemagglutination and the 50% tissue culture infection dose (TCID50) assay, and malealdehyde (MDA) analysis collaborated with transmission electron microscopy (TEM) was used to analysis of lipid peroxidation in the viral envelope, and western blot were applied to detect the adjacent viral proteins. Ionzymes was demonstrated to be able to catalyze lipid peroxidation of the viral lipid envelope to inactivate the enveloped virus and provide protection from viral transmission and infection, verifying the inactivation of influenza A virus (IAV). Yang group developed a nitrogen and iodine-doped carbon spot (I-CD) as a peroxidase-mimic (POD) nanozyme for antifungal disinfection of C. albicans (Li X et al., 2021). In the existence of exogenous H_2_O_2_, the prepared I-CDS samples presented a peroxidase activity, and the antifungal activity against *Candida* albicans was up to 90%. The system promoted the formation of ROS, containing hydroxyl radical (·OH), which successfully inhibited the growth of *Candida* albicans. Nanozyme has been an ideal substitute for antibiotics for inhibiting resistant bacteria thanks to the mechanism of its action and broad spectrum and durability.

### Inflammation Elimination

**FIGURE 7:**
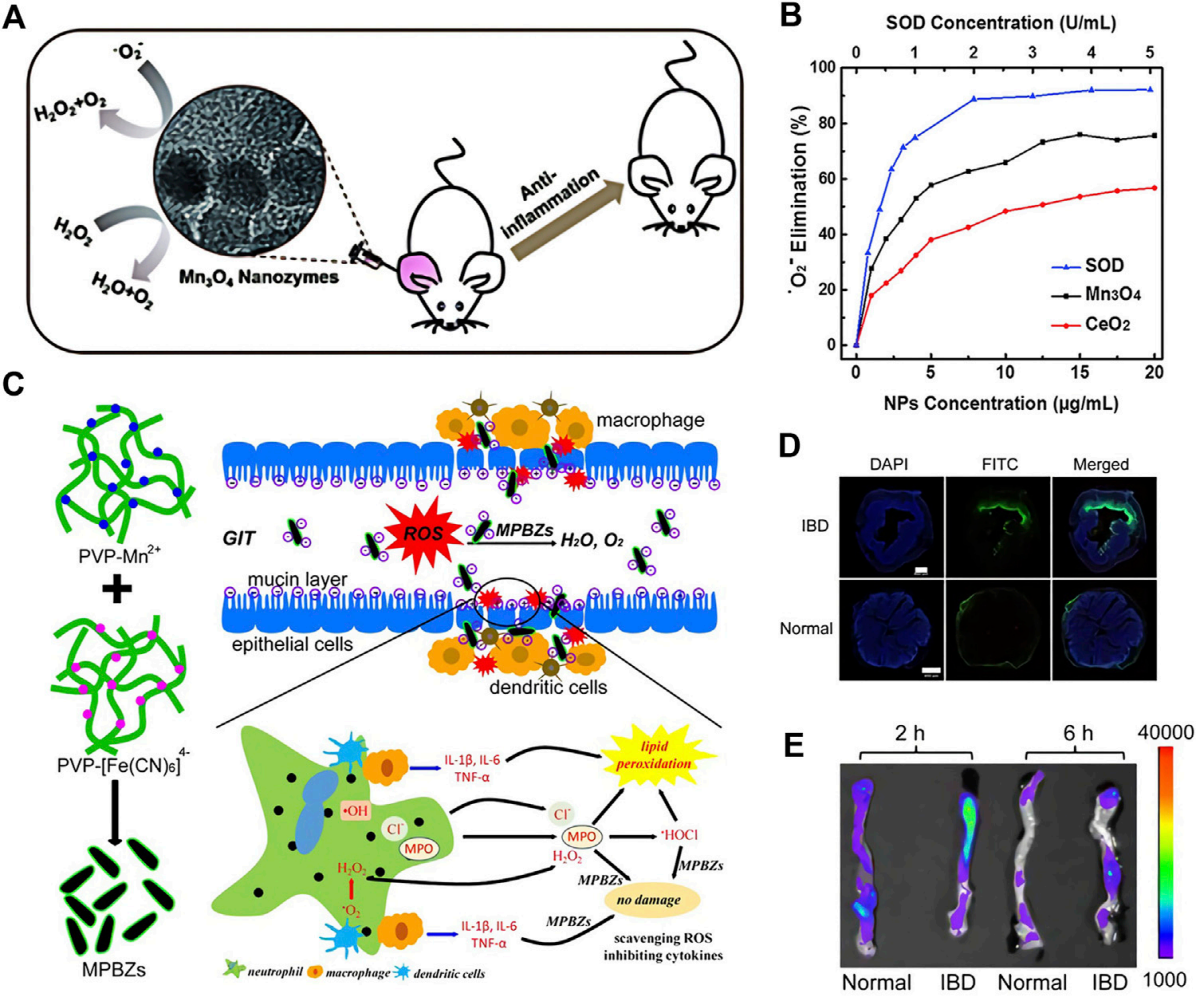
The schematic diagram of Mn_3_O_4_ nanozymes **(A)**. Dependence between the elimination of O_2_- and concentrations of Mn_3_O_4_ NPs, CeO_2_ NPs, and natural SOD **(B)**. Schematic diagram of the synthetic production of MPBZs. MPBZs modified with PVP were constructed via a simple but efficient strategy: mixing the manganese source solution with PVP and an [Fe(CN)^6^]_4_- source with PVP under magnetic stirring **(C)**. Fluorescence imaging of colons from control mice and mice with DSS-induced colitis after oral FITC-labelled MPBZ treatment **(D)** Biodistribution images and in the colons of mice treated with MPBZs were carried out after oral administration of 2 and 6 h. Scale bar: 400 μm **(E)**. (**A,B** was reported with permission from Ref. (Yao et al., 2018) Copyright 2018 Royal Society of Chemistry. **C–E** was reported with permission from Ref. (Zhao et al., 2019) Copyright 2019 Ivyspring International.)

Inflammation is a normal immune response. Under normal circumstances, inflammation is beneficial and belongs to the automatic defense mechanism of body. But if it cannot be stopped in time, acute inflammation will develop into chronic inflammation or even cancer. The occurrence of inflammation is inseparable with excessive ROS production. Therefore, antioxidants are extremely significant in the treatment of inflammation (Poole et al., 2015). On this ground, Wei et al. prepared Mn_3_O_4_ nanoparticles, which own both CAT and SOD activities, and possess a clear effect on O_2_·−, H_2_O_2_ and •OH (Figures 7A,B) (Yao et al., 2018). The animal experiment results exhibited that Mn_3_O_4_ nanoparticles can effectively alleviate ear inflammation caused by abnormally elevated ROS in living mice. Besides, Hyeon et al. (Soh et al., 2017) prepared Ce_0.7_Zr_0.3_O_2_ nanoparticles that by introducing Zr^4+^, the nanozyme were endured with a higher Ce^3+^/Ce^4+^ ratio and accelerate the conversion speed of Ce^4+^ to Ce^3+^.In comparison with CeO_2_ nanoparticles alone, the ROS scavenging ability of the nanozyme is significantly promoted, which can regulate the inflammatory response at very low doses. The results demonstrated that in the two typical sepsis model, Ce_0.7_Zr_0.3_O_2_ is capable of lowering the mortality and systemic inflammation in mice by 60%.

Except for eliminating ROS draw support with catalytic reactions, nanozymes is able to regulate the immune system and alleviate inflammation. Zheng et al. (Figures 7C–E) (Zhao et al., 2019) synthesized Prussian blue manganese nanozymes (MPBZs) with a variety of enzymatic activities. With its suitable size (120 nm) and negative charge effect, nanozyme can preferentially accumulate on the mucosal surface after oral administration.

Xiong et al. found that Fe_2_O_3_ dimercaptosuccinate nanoparticles can protect the heart from ischemic injury (Xiong et al., 2015). Fe_2_O_3_ NPs increased the activity of SOD and decreased the level of ROS when heart ischemia-reperfusion injury occurred. Zhang et al. (2016). demonstrated that PB NPs have been proved to possess SOD and CAT activities, and revealed that PB NPs can be an effective antioxidant to control ROS-induced cell damage. *In vitro* experiments verified that ROS levels increased in rat mesangial cells (HBZY-1 cells) stimulated by CDDP. However, PB NPs effectively inhibited ROS levels and increased cell viability. Based on these results, it is suggested that PB NPs may potentially inhibit the nephrotoxicity of CDDP. The antioxidant effect of PB NPs has also been confirmed *in vivo* inflammation experiments.

Gold nanozyme can also down-regulate ROS. Barathmanikanth et al. found that SOD activity was significantly enhanced in the diabetic treatment group treated with Au NPs compared with the diabetic control group (Barathmanikanth et al., 2010). The ability of Au NPs to reduce ROS levels in hyperglycemia is able to restore the antioxidant defense system, showing the prospect of inflammation elimination.

## Summary and Outlook

Nanozymes are a class of nanomaterials with enzymatic activity. In comparison with natural enzymes, nanozymes own the merits of flexible composition design, high stability, simple preparation and adjustable catalytic activity. In this review, the application of nanozyme in the diseases therapy in recent years is summarized, and the three strategies of nanozyme in the treatment of diseases are introduced in detail. Although nanozymes have made up for many shortcomings of traditional pro-oxidants/antioxidants and are widely applied in the field of disease treatment, there are still a sea of problems needs to be counter urgently.

1) Catalytic activity: Compared with natural enzymes and organic catalysts, the catalytic activity and efficiency of nanozymes are relatively low, which severely limits their application in disease treatment. On this ground, there is an urgent need to develop nanozymes with high catalytic activity, to further improve the therapeutic effect. According to the characteristics of nano-enzyme, the activity of nano-enzyme can be promoted by adjusting its size, crystal morphology, composition, surface modification, and the construction of mixed nano-enzyme. In addition, the current research on nano-enzymes mainly focuses on the simulation of oxidoreductase and hydrolase, while the simulation of ligase and transferase, which also play a significant role in living organisms, is still unknown. Therefore, expanding the types of nanozyme will be the only way to expand the application of nano-enzyme. In order to achieve this goal, it is essential to possess a full understanding of the enzyme activity center of the natural enzyme, and then combine the existing synthetic methods to simulate the active center and assemble the material to realize the simulation of the catalytic activity. In terms of enzyme activity, another key issue is the regulation between the activities of multiple enzymes of nanozyme. At present, nanozyme mainly realizes the treatment of various diseases through its positive or reverse regulation of ROS. However, according to the above, a nanozyme often has multiple enzyme activities, so how to balance its antioxidantase-like enzyme and oxidase-promoting enzyme activities is of great significance to ensure accurate and stable therapeutic effects. Constructing environment-responsive nanozyme or modifying the surface of nanozyme are effective measures to solve this problem.
2) Safety: Although nanozymes present great potential in disease treatment, in order to truly develop into clinical applications in the future, research on their biosafety *in vivo* is essential. Different from traditional enzymes, nanozymes are mostly inorganic nanoparticles, which can enter lysosome, mitochondria or nucleus in cells, and may cause damage to cells by irreversible interaction with intracellular components. In addition, as a foreign substance, nanozymes may also lead to immune responses in the body. To address this problem, studies have demonstrated that coating the surface of nanoparticles with polymers such as polyethylene glycol, cell membrane to promote their biocompatibility. However, the ideal biocompatibility is still a challenge.
3) Particle Size: The blocked metabolism of nanoparticles in the body can also trigger potential body damage. Studies have confirmed that nanoparticles smaller than 10 nm tend to accumulate in bone marrow or be removed by kidney, while nanoparticles larger than 100 nm tend to accumulate in macrophages of liver or spleen and cause hepatotoxicity (Alexis et al., 2008; Jun et al., 2008). Therefore, designing nanozymes of reasonable size to achieve ideal results and reduce side effects will be the focus of future research.
3) Mechanism of Resistance to disease: To date, the mechanism of action of many nanozymes in disease treatment remains unclear. As an example, it has been reported in some literatures that the intracellular ROS level decreased and the tolerance to oxidative stress increased after nanoparticles were applied to cells (Li Y et al., 2015). Unfortunately, there is no conclusive evidence from these studies that the antioxidant enzyme activity of nanozyme plays a role in cell protection.
4) Other potential applications: micro/nanomotors, as an emerged new research aspect, attract global interests due to their great potential applications in environmental remediation and biomedical field. Enzymes, with intrinsic high catalytic ability and excellent biocompatibility, are nice candidates for the engines of micro/nanomotors (Chen H et al., 2020; Wang Y et al., 2019; Wang et al., 2020). However, the natural enzymes are also facing the limitations for harsh environmental influence, where nanozymes would address the problem with their advantages, thus could further facilitate the development of micro/nanomotors, towards deep applications in biomedical fields.

In conclusion, the development of nanozymes with high enzyme activity, superior stability, selective specificity, excellent biosafety and applicability, as well as the further study of their catalytic mechanism to achieve the expansion of indications will be the research focus of the application of nanozymes in the field of disease treatment in the future.
